# Fault Diagnosis in Centrifugal Pumps: A Dual-Scalogram Approach with Convolution Autoencoder and Artificial Neural Network

**DOI:** 10.3390/s24030851

**Published:** 2024-01-28

**Authors:** Wasim Zaman, Zahoor Ahmad, Jong-Myon Kim

**Affiliations:** 1 Department of Electrical, Electronic and Computer Engineering, University of Ulsan, Ulsan 44610, Republic of Korea; wasim94@mail.ulsan.ac.kr (W.Z.); zahooruou@mail.ulsan.ac.kr (Z.A.); 2 Prognosis and Diagnostics Technologies Co., Ltd., Ulsan 44610, Republic of Korea

**Keywords:** centrifugal pump, Stockwell transform, fault diagnosis, rotating machinery, convolutional autoencoder, vibrational signals, deep learning

## Abstract

This paper proposes a new fault diagnosis method for centrifugal pumps by combining signal processing with deep learning techniques. Centrifugal pumps facilitate fluid transport through the energy generated by the impeller. Throughout the operation, variations in the fluid pressure at the pump’s inlet may impact the generalization of traditional machine learning models trained on raw statistical features. To address this concern, first, vibration signals are collected from centrifugal pumps, followed by the application of a lowpass filter to isolate frequencies indicative of faults. These signals are then subjected to a continuous wavelet transform and Stockwell transform, generating two distinct time–frequency scalograms. The Sobel filter is employed to further highlight essential features within these scalograms. For feature extraction, this approach employs two parallel convolutional autoencoders, each tailored for a specific scalogram type. Subsequently, extracted features are merged into a unified feature pool, which forms the basis for training a two-layer artificial neural network, with the aim of achieving accurate fault classification. The proposed method is validated using three distinct datasets obtained from the centrifugal pump under varying inlet fluid pressures. The results demonstrate classification accuracies of 100%, 99.2%, and 98.8% for each dataset, surpassing the accuracies achieved by the reference comparison methods.

## 1. Introduction

Centrifugal pumps (CPs) are crucial components across a range of industries, including oil processing, mineral extraction, and power generation. A survey by major organizations revealed that an investment of 10,000–20,000 USD in health management can lead to annual savings of up to 500,000 USD [1]. This demonstrates the significant financial benefits of proactive maintenance strategies. A pump failure can cause operational delays, interrupt production, and may even lead to dangerous accidents, leading to financial losses and potentially drastic outcomes, like company bankruptcy or a drop in stock prices [2,3]. Recognizing the severe implications of pump malfunctions, the diagnosis and prompt handling of faults in centrifugal pumps become critical concerns.

Faults typically arise in components like bearings, mechanical seals, or impellers, with the latter two often being overlooked in research despite their significance [3]. While bearings have been extensively studied [4,5], the literature on mechanical seal and impeller faults is not as comprehensive. This gap underscores the need for an inclusive diagnostic framework that addresses these components, enhancing pump reliability and ensuring industrial safety [6]. Our research aims to fill this void, proposing a method that considers the complexities of mechanical seal and impeller defects to improve the dependability of centrifugal pumps.

Mechanical seal faults notably influence the vibration signals from a CP, making them appear impulsive and nonstationary. This complicates the analysis of these vibration signals for diagnosis purposes [7]. To clearly identify the faults, these vibration signals need to be preprocessed. This is essential, as the features indicating faults are often masked by significant noise and fading impulses in the signals. The fundamental approaches for signal processing fall under three categories: time, frequency, and time–frequency domain (TFD) analyses.

Vibration shifts from MFs in CPs can lead to changes in the signal amplitude and distribution [8]. Time domain statistical features capture these shifts [9,10,11] but are not sensitive enough to fault severity changes. In contrast, the frequency spectrum is more responsive to faults, showing distinct spectral lines corresponding to changes [12]. However, the vibration signals from CPs are largely nonstationary, and Fourier transforms (FTs) are mostly suited for stationary signals [13,14]. Time–frequency methods like the wavelet transform (WT) have been applied in recent years to address this nonstationary behavior [15,16], but finding the optimal wavelet function remains challenging. Empirical mode decomposition (EMD) has been introduced to address the limitations of the wavelet transform [17], and while effective, it also has its limitations [18,19,20].

Yan et al. [21] proposed an optimal wavelet selection method for defect identification in rotating machinery based on the energy content of wavelet coefficients, followed by a Fourier transform on feature envelopes. Delgado et al. [9] utilized statistical time features from vibration signals, applied nonlinear manifold learning for dimensionality reduction, and employed a hierarchical neural network for bearing fault diagnosis. In 2017, Xia et al. [22] presented a CNN-based approach for rotating machinery diagnosis, merging vibration data from various sensors into a 2D matrix and training a CNN on these features. Maamar et al. [23] introduced a novel method for centrifugal pump fault diagnosis in 2019 that integrated a genetic algorithm with artificial intelligence. Jing et al. [24] established a novel algorithm for spalling propagation assessment in bearings, leveraging spectrum amplitude ratios and statistical features to pinpoint the spalling damage location and severity. In 2020, He et al. [25] introduced a novel hybrid deep signal processing method for bearing fault diagnosis, which combined vibration analysis techniques with deep learning, and embedded a time-synchronous resampling mechanism. Ahmad et al. [26] developed a three-stage technique in 2021: first, using the Walsh transform on CP vibration signatures, then extracting statistical features, and finally applying cosine linear discriminant analysis (CLDA) for fault classification using the KNN algorithm. Another method by Ahmad et al. [27] for diagnosing faults in multistage centrifugal pumps (MCPs) involved using informative ratio principal component analysis (Ir-PCA) on features from the vibration signal’s fault-specific frequency band, which was then classified using the KNN algorithm. Cabrera et al. [28] developed an innovative fault detection model, designed for cyclo-stationary machines with limited data, and initially employed an unsupervised generative adversarial network (GAN) to model vibration signals. Siddique et al. developed a novel hybrid technique that merges the STFT and CWT scalograms, aiming to advance the detection of leaks in pipeline systems [29]. Ullah et al. [30] introduced a fault diagnosis method for CPs that leverages wavelet coherence analysis and deep learning to enhance fault detection. In 2023, Zaman et al. [31] ntroduced a pioneering framework for classifying centrifugal pump conditions, combining signal processing and deep learning, with a key contribution being the development of innovative SobelEdge scalograms.

While the literature demonstrates an improved performance in CP fault diagnosis, it also reveals significant shortcomings: firstly, the challenging task of mother wavelet selection for wavelet transformations and, secondly, an industrial requirement for a generic method applicable to most datasets, given that these methods exhibit efficacy for one dataset but may not perform as well with others. Addressing the challenges, we introduce a novel approach that merges signal processing and deep learning methodologies: the vibration signal is first processed through a lowpass filter to isolate fault-specific frequencies and then subjected to two parallel transformations—a continuous wavelet transform (CWT) and a Stockwell transform—resulting in two distinct scalograms, from which features are extracted via a convolutional autoencoder (CAE). Subsequently, features from both parallel pipelines are amalgamated into a robust feature pool, which is fed into an artificial neural network (ANN) for classification into distinct fault categories. The main contributions of our method are bulleted below:

(1) Integration of signal processing and deep learning: This approach uniquely combines traditional signal processing techniques with deep learning methods to enhance the analysis and classification of vibration signals.
(2) Parallel transformations: The use of two different transformations—the CWT and Stockwell transforms—enables the capture of different yet complementary information from the vibration signal. This dual transformation approach is a distinctive feature of this work.
(3) Scalogram feature extraction via CAE: A CAE is employed to extract relevant features from the scalograms resulting from the CWT and Stockwell transforms. Using a CAE ensures that only the most pertinent features, which might otherwise be hard for traditional methods to capture, are retained.
(4) Amalgamation of features: Features extracted from both the CWT and Stockwell scalograms are combined into a single robust feature pool, which is expected to offer a comprehensive representation of the vibration signal.
(5) ANN-based classification: The consolidated feature pool is then input into an ANN for effective classification into distinct fault categories. This demonstrates the applicability of deep learning in making sense of the complex features derived from vibration signals.

The present study is organized as follows: Section 2 presents a detailed discussion of the materials and methods, while Section 3 describes the experimental setup and the test rig setup for the CP. Section 4 provides results discussion and evaluation metrics, and, finally, Section 5 provides a summary of the findings and proposes recommendations for future research.

## 2. Materials and Methods

### 2.1. Technical Background

The subsequent sub-sections provide an in-depth exploration of select technical terminology.

#### 2.1.1. Continuous Wavelet Transform Scalograms

In the realm of diagnosing faults in rotating machinery like centrifugal pumps, time domain and frequency domain analyses are frequently employed. Yet, these methods have a limitation: they do not simultaneously illustrate the signal’s variations across both time and frequency domains. It is crucial to note that, in real-world scenarios, the signals derived from centrifugal pumps often exhibit nonstationary and nonlinear characteristics [5,32]. Consequently, time–frequency analysis emerges as a potent tool, adeptly unveiling the concealed fault information within centrifugal pump signals, presenting them visually as images [33]. The time–frequency domain methodology dissects signals across varied time and frequency scales, subsequently presenting them as a two-dimensional visual representation [34]. This image encompasses both the local (pertaining to specific segments of the image) and global (encompassing the entire image) traits of the signal. Importantly, these two-dimensional depictions serve as robust input features for deep learning algorithms, thereby enhancing their diagnostic precision. Given these advantages, it is advantageous to adopt the time–frequency technique when analyzing vibration signals from centrifugal pumps.

In this study, we harness the capabilities of the CWT to analyze pump signals. At its core, the CWT employs a set of mother wavelet functions, transforming the input signal into a defined spectrum of wavelet coefficients. This transformation is systematically achieved by both translating and scaling the foundational signal across varied time and frequency domains. Notably, when faults arise, they generate a diverse range of frequencies, from low to high, within the initial signal. This results in a fluctuating energy distribution across multiple frequency spectrums. By utilizing the CWT, we can adeptly redistribute the energy from the primary signal across its decomposed variants, effectively pinpointing those time–frequency scales laden with significant energy. Consequently, these wavelet coefficients become instrumental, offering valuable insights into the health metrics of mechanical components, as substantiated by reference [35]. A more granular breakdown of the CWT methodology is further delineated in the following mathematical representations:

(1) $$W\left(a,b\right)={\left|a\right|}^{-\frac{1}{2}}\psi \;(\frac{t-b}{a})$$

where a,b∈R and a≠0.

The coefficient $C_{a}(k)$ can be computed as follows:

(2) $$C_{a}\left(k\right)=\int x\left(t\right)\psi_{a,b}\left(t\right)dt$$

Consider $\psi_{a,b}(t)$ as the mother wavelet function. Its form and positioning are determined by two parameters: the scale, represented by a, and the location or translation, signified by b. The signal in its original form is denoted by x(t). As we move through different scales, specifically from 1 to l, the wavelet coefficients can be identified as $C_{a}$. Additionally, $\psi_{a,b}(t)$ represents the complex conjugate of $\psi_{a,b}\;(t)$ at a specified scale a and position b. This results in a fluctuating energy distribution across multiple frequency spectrums. By utilizing the CWT, we can adeptly redistribute the energy from the primary signal across its decomposed variants, effectively pinpointing those time–frequency scales laden with significant energy.

In the realm of CWT methodologies, the Morlet wavelet stands out as a preferred choice due to its inherent properties [36]. This preference is further accentuated by the introduction of the scalogram, which serves as a visual representation of the CWT coefficients across varying scales (p,q) [35]. A scalogram maps these coefficients onto a two-dimensional time–frequency domain, where the *x*-axis signifies the translation or temporal aspect and the *y*-axis delineates the scale parameter, inversely related to the signal’s frequency. Notably, the color intensity of each pixel within the scalogram is directly linked to the magnitude of the corresponding wavelet coefficient. This representation vividly showcases how the energy of the initial signal spreads across the time–frequency continuum [37]. Consequently, the CWT not only captures localized energy fluctuations across distinct regions of this time–frequency representation but also positions the CWT scalograms (CWTSs) as potent discriminators. These scalograms, through their translation of wavelet coefficients into time–frequency imagery, hold the capability to discern the varied health states of a centrifugal pump.

#### 2.1.2. Stockwell Transform Scalograms

The S-transform adeptly integrates elements of both the short-time Fourier transform (STFT) and the wavelet transform, positioning it as an advanced time–frequency spectral localization technique. This transformative method emerges primarily as a “phase correction” derivative of the CWT. Within this context, the CWT, represented as T(τ, d), is intrinsically tied to the function g(t), as delineated by its mathematical definition.

(3) $$T(\tau ,d)=\int_{\infty }^{-\infty }g(t)\omega (t-\tau ,d)dt$$

Here, ω(t,d) denotes a scaled iteration of the foundational mother wavelet. The dilation parameter, d, dictates the resolution and essentially specifies the “breadth” of the wavelet ω(t,d). Beyond the stipulations of Equation (1), it is imperative for the mother wavelet ω(t,d) to satisfy the admissibility condition, ensuring it maintains a zero mean, as highlighted in [38].

Per the definition, the S-transform of function h(t) is a CWT that uses a distinct mother wavelet, scaled by a phase factor:

(4) $$S(\tau ,f)=e^{i2\pi f\tau }T(\tau ,d)$$

The designated mother wavelet is characterized as follows:

(5) $$\omega (\tau ,f)=\frac{\left|f\right|}{\sqrt{2\pi }}e^{-\frac{t^{2}f^{2}}{2}}e^{-i2\pi f\tau }$$

It is crucial to observe that the frequency f and the dilation factor d are reciprocal to each other. Given that the wavelet in Equation (3) does not align with the stringent criteria of a CWT (having a zero mean), Equation (2) cannot be considered a legitimate CWT.

To define the S-transform in mathematical terms, consider the subsequent formula:

(6) $$S(\tau ,f)=\int_{\infty }^{-\infty }g\left(t\right)\frac{\;\left|f\right|}{2\pi }e^{-\frac{{\left(\tau -t\right)}^{2}f^{2}}{2}}\;e^{i2\pi f\tau }dt$$

When representing the local spectrum via the S-transform, one should be able to derive the Fourier spectrum by simply averaging these local spectra over time [39].

Utilizing the S-transform, a signal is partitioned into sequential time–frequency segments. Each of these segments elucidates the energy distribution of the signal for a specific frequency over a short time span. From these segments, scalograms can be generated, visually representing the signal’s energy distribution across both the time and frequency domains. These scalograms are created by mapping the intensity of each time–frequency segment to a color scale, thereby producing a two-dimensional image that illustrates the variations in signal energy over time and frequency [39]. Within the S-transform of a vibration signal, brighter regions signify a heightened energy content at a particular frequency during a defined time frame. To enhance the visibility of these brighter zones, we applied the Sobel filter edge extraction technique. This method effectively highlights the edges within the scalogram, thereby emphasizing areas with significant energy shifts, which are crucial for a more accurate analysis and interpretation of the signal’s characteristics.

**Figure 1:**
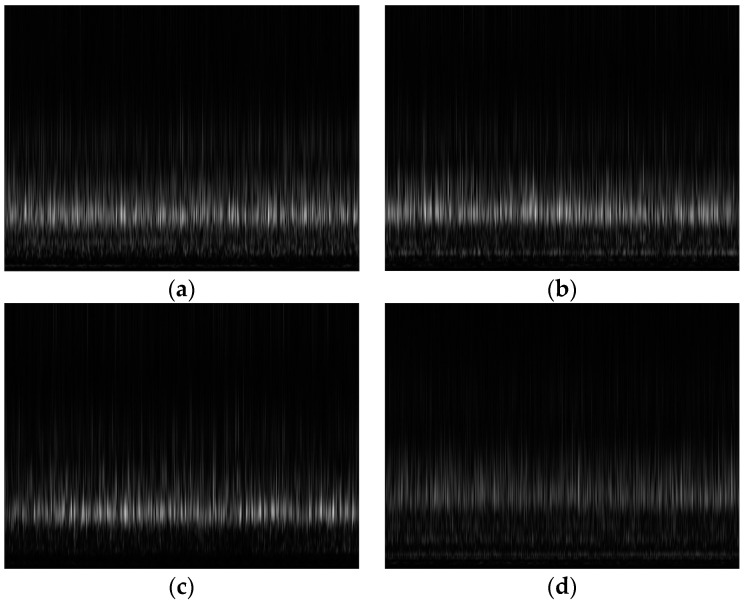
Sobel-filtered Stockwell transform scalograms of (**a**) impeller fault, (**b**) mechanical seal hole, (**c**) mechanical seal scratch, and (**d**) normal conditions.

Within the S-transform, the more luminous areas serve as indicators, pinpointing segments of a vibration signal characterized by greater amplitudes or pronounced vibrations. This information becomes instrumental in scrutinizing and discerning the underlying system dynamics responsible for the vibration signal. Notably, when there was a shift in the operating conditions of the CP, the scalograms derived from its vibration signals using the S-transform vividly showcased distinct luminous regions. The respective S-transform scalograms of the vibration signals for every operating state of the CP are illustrated in Figure 1.

#### 2.1.3. Sobel Filter

In the realm of image processing, the Sobel filter stands out as an essential tool for edge detection. This filter operates by superimposing an image with a compact matrix, known as the Sobel operator. Intriguingly, this operator computes the gradient at individual pixels, leading to an amplified delineation of edges within the image. Venturing into the context of Stockwell scalograms, one observes the Sobel filter’s adaptation as a digital tool tailored for the time–frequency representation intrinsic to these two-dimensional signals. To achieve this scalogram, the S-transform is employed, wherein an intricate Fourier transform is applied over a continuum of signal segments. This is further succeeded by computing the resultant spectrum’s magnitude. A key aspect of the Sobel filter’s utility lies in its comparison of adjacent frequency components within the scalogram. This facilitates the discernment of abrupt shifts in the frequency domain of the signal. Such nuances can often hint at critical events embedded in the signal, be they transients or anomalies. Such insights hold paramount significance in specialized domains, notably in applications like leak detection and condition monitoring [40].

In the domain of signal processing, the implementation of a Sobel filter for Stockwell scalograms often hinges on the specific needs of the application and the desired outcome. Generally, this filter operates by juxtaposing neighboring frequency components within the scalogram, aiming to pinpoint sudden fluctuations in the signal’s frequency spectrum. Once filtered, the resultant scalogram provides a richer canvas for analysts to scrutinize specific regions or features that might be indicative of anomalies, such as potential leaks in a fluid system [41].

The efficacy of the Sobel filter is closely tied to three critical factors: the integrity of the S-transform, the meticulously crafted design of the filter itself, and, crucially, the expertise of the user in deciphering the post-filtered scalogram. Encompassing these attributes, Sobel filters tailored for Stockwell scalograms emerge as an indispensable instrument in discerning alterations in signal frequencies. Their versatility underscores their relevance across a broad spectrum of signal processing and condition monitoring endeavors [42].

#### 2.1.4. Convolution Autoencoder

The CAE seamlessly integrates local convolution connections and traditional autoencoders, introducing a reconstruction facet to the convolutional process. This transformation of feature maps from input to output is termed the “convolutional decoder”. Subsequently, using an inverse convolutional approach—referred to as the “convolutional encoder”—these outputs are reshaped. Utilizing the foundational unsupervised greedy training inherent to autoencoders, it becomes feasible to compute the parameters for both encoding and decoding operations [43].

**Figure 2:**
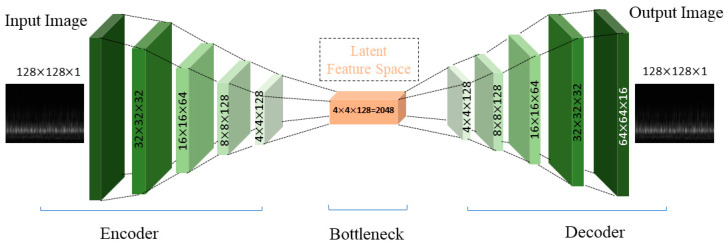
Architecture of the convolutional autoencoder.

The workings of the convolutional autoencoder layer are depicted in Figure 2. Here, f(.) denotes the convolutional encoding function, while $f^{\prime }(.)$ symbolizes the decoding counterpart. The input consists of feature maps $x\;\epsilon \;R^{n\times l\times l}$, either sourced from the initial layer or the preceding one. This input comprises *n* feature maps, each spanning an area of l×l pixels. The convolutional autoencoder operation encompasses m convolutional kernels, producing m feature maps in the output layer. If these feature maps stem from the input layer, n designates the count of input channels. However, if they are derived from a prior layer, n signifies the total output feature maps of that preceding layer. The convolutional kernel’s dimensions stand at d×d, ensuring d≤l.

The set of parameters $\theta =\{W,\hat{W},b,\hat{b},\}$ defines the learning elements of the convolutional autoencoder layer. Among these, $b\in R^{m}$ and $W=\{w_{j},j=1,2,...,m\}$ correspond to the parameters of the convolutional encoder. Here, each $w_{j}\in R^{n\times l\times l\;}$ can also be represented as a vector $w_{j}\in R^{nl^{2}}$. On the other side, $W=\{w_{j},j=1,2,...,m\}$ and $\hat{b}$ are the parameters for the convolutional decoder. For these, $\hat{b}\in R^{nl^{2}}\;$and each ${\hat{w}}_{j}\in R^{1\times nl^{2}}$.

Initially, the input image undergoes an encoding process. During this phase, patches of size d×d pixels, denoted as $x_{i}$ where i=1,2,...,p, are extracted from the input image. Subsequently, for each patch, the weight $w_{j}$ of the $j^{th}$ convolution kernel is employed for convolution operations. This results in the computation of the neuron values $o_{ij}$ for j=1,2,...,m in the output layer:

(7) $$o_{ij}=f\left(x_{i}\right)=\sigma \left(w_{j}\cdot x_{i}+b\right)$$

where σ represents a nonlinear activation function. In the current study, the rectified linear unit activation function is utilized.

(8) $$Relu(x)=\left\{\begin{array}{c} x\;x\geq 0\\ 0\;x<0 \end{array}\right.$$

Following this, the $o_{ij}$ output from the convolutional decoder undergoes encoding, wherein $x_{i}$ is reconstructed using $o_{ij}$ to produce ${\hat{x}}_{i}$.

(9) $$x_{i}=f^{\prime }(o_{ij})=\phi (w_{i}\cdot o_{ij}+\hat{b})$$

Following the convolutional encoding and decoding processes, ${\hat{x}}_{i}$ is generated for each instance. From the reconstruction operation, we obtain P patches, each of size d×d. The cost function is defined as the mean square error between the original patch of the input image $x_{i}$ (where i=1,2,…,p) and the reconstructed patch ${\hat{x}}_{i}$ (where i=1,2,…,p). The specific form of the cost function is presented in Equation (10), while the reconstruction error is detailed in Equation (11) [43].

(10) $$J_{CAE}(\theta )=\frac{1}{p}\sum_{i=1}^{p}L[x_{i},{\hat{x}}_{i}]$$

(11) $$L_{CAE}\left[x_{i},{\hat{x}}_{i}\right]=∥x_{i}-{\hat{x}}_{i}{∥}^{2}=∥x_{i}-\phi \left(\sigma \left(x_{i}\right)\right){∥}^{2}$$

Utilizing stochastic gradient descent (SGD), the weights and errors are iteratively refined, leading to the optimization of the convolutional autoencoder layer. Once trained, these optimized parameters are employed to produce the feature maps, which are then forwarded to the subsequent layer.

In the current study, the CAE model is meticulously designed with multiple layers, each serving a specific function in the process of encoding and decoding the input data. The model starts with an input layer that receives the scalogram, which is then passed through successive convolutional layers. These layers progressively reduce the dimensionality of the image, isolating key features. Following the encoding process, the model transitions to the decoding phase, where it reconstructs the image back to its original form, albeit now enriched with extracted features. This reconstructed output is crucial for identifying and classifying various fault conditions in the pumps. Table 1 illustrates the CAE layers and their output dimensions, which are utilized in the current study.

#### 2.1.5. t-SNE Analysis

The machine learning technique known as t-SNE (t-distributed stochastic neighbor embedding) is often utilized to visualize high-dimensional data in a 2D or 3D scatter plot. The technique preserves the local structure of the data, ensuring that similar points in the high-dimensional space are positioned closely in the low-dimensional space [44]. This visualization facilitates the discovery and interpretation of patterns and structures within complex datasets.

In our research, we employed t-SNE to visualize latent space representations obtained from an autoencoder. This dimensionality reduction technique projects complex, high-dimensional features into a 2D space, enabling analysis of feature entropy and data clustering. Such visualization uncovers latent feature distributions and structures, providing valuable insights for interpreting encoded data representations. The t-SNE application reveals distinct clusters, indicative of diverse states or categories, enhancing our understanding of the feature space organization. This insight is instrumental in refining the autoencoder architecture and optimizing feature extraction parameters, thereby improving the model performance in complex pattern recognition tasks, like anomaly detection and sophisticated classification.

#### 2.1.6. Hyperparameter Selection

Hyperparameter tuning is essential in optimizing deep learning model performance, ensuring accurate and efficient results. In our study, we systematically fine-tuned various model aspects, such as the batch size, learning rate, optimizer type, number of hidden layers, and training epochs to enhance the learning capability and generalization performance. Additionally, we utilized t-SNE for effective feature visualization, which was pivotal in achieving a meaningful representation of the high-dimensional feature space in a reduced dimensional format. Key t-SNE parameters included a perplexity value of 30 and a learning rate of 200, selected to maintain a balance between local and global data aspects, thus ensuring accurate data representation. The specific values and settings for all these hyperparameters, iteratively refined through multiple experiments, are detailed in Table 2. These hyperparameters, particularly those of t-SNE, have been instrumental in visualizing feature vectors, thereby significantly enhancing the interpretability of the model’s feature extraction capabilities.

### 2.2. Proposed Approach

Fault diagnosis in rotating machinery utilizing vibration signals often employs the CWT and the S-transform. These time–frequency signal analysis techniques are favored for their proficiency in capturing the nonstationarity of signals. Particularly for rotating components like impellers and mechanical seals, these transformations prove invaluable in CP fault diagnosis. They adeptly identify mechanical faults, such as holes or scratches in mechanical seals and impeller faults, and also distinguish between the healthy and faulty states of the CP. However, it is noteworthy that the scalograms derived from these transformations can occasionally retain some noise.

The edges play a pivotal role in both the CWT and S-transform scalograms. To accentuate these edges, the Sobel filter is applied across the scalograms. When both types of scalograms undergo the Sobel filter edge detection process, the visibility of the edges within the time–frequency scalograms is significantly enhanced. Furthermore, this refined edge detection proves beneficial for the CAE, enabling it to extract more discerning features with ease.

**Figure 3:**
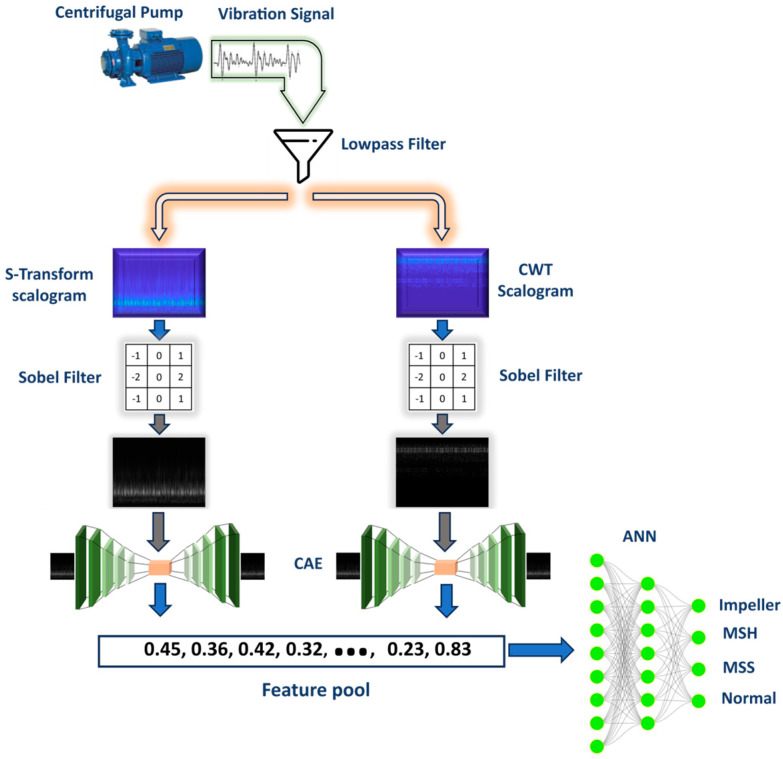
Flowchart of the proposed approach.

In the proposed methodology, we integrate signal processing and deep learning techniques for fault detection in CPs. Initially, vibration signals are acquired using sensors affixed to the CP. During preprocessing, a lowpass filter with a cutoff frequency set at 4.6 kHz is employed to isolate fault-specific frequencies. Subsequently, these isolated frequencies are concurrently subjected to the CWT and the S-transform, resulting in two distinct time–frequency scalograms for each vibration signal. To mitigate noise and accentuate pertinent details, these scalograms are processed using the Sobel filter, which emphasizes the edges. For optimal feature extraction, we deployed two parallel CAE models tailored for each scalogram type. Extracted features from both scalograms are then amalgamated into a comprehensive feature pool, characterized by its discriminative properties conducive for classification. As the culmination of this pipeline, a two-layer ANN is trained on this feature pool to achieve fault classification. The entire workflow is succinctly depicted in Figure 3.

The outlined approach can be succinctly described through the following structured steps:

(1) Data acquisition: Gather vibration signals from the CP under various health scenarios using a specialized data acquisition system.
(2) Signal preprocessing: Implement a lowpass filter with a cutoff frequency of 4.6 kHz to isolate and capture fault-specific frequencies from the signals.
(3) Time–frequency analysis: Simultaneously produce two distinct scalograms through the CWT and S-transform methods.
(4) Edge enhancement: Apply the Sobel filter on the scalograms to accentuate edges, thereby mitigating noise.
(5) Feature extraction: Utilize the refined scalograms to train two parallel CAEs, subsequently extracting pertinent features from each.
(6) Combining features: Integrate the extracted features from both CAEs into a comprehensive and discriminative feature pool.
(7) Classification: Train an ANN on this feature pool, enabling precise classification across diverse CP health conditions.

## 3. Experimental Methods and Test Rig Setup

**Figure 4:**
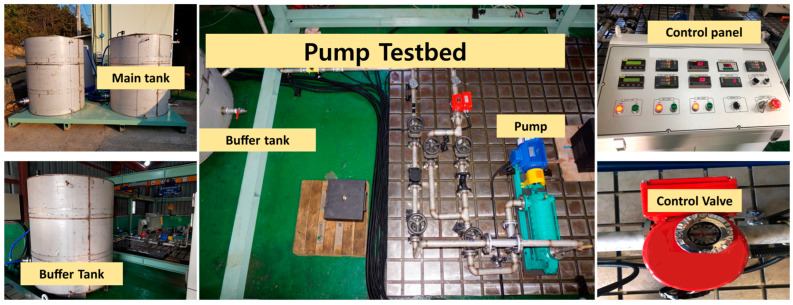
Setup for vibration data acquisition experiment [45].

**Figure 5:**
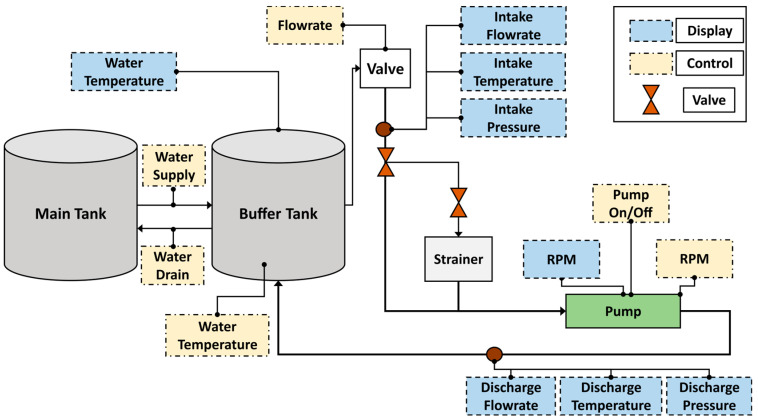
Illustrative layout of the experimental test rig [45].

In this study, we conducted experiments utilizing a CP, specifically the PMT-4008 model, which is prevalent in industrial settings. This pump was powered by a 5.5 kW motor. Accompanying the pump, we had a control panel outfitted with various elements: an ON/OFF switch; controllers for the speed, flow rate, temperature, and water supply; display screens; and pressure gauges. Our setup also included clear steel pipes and two tanks–a main tank and a buffer tank. To ensure the consistent operation of the CP, the water tank was strategically placed at an elevation that would maintain the net positive suction head (NPSH) at the pump’s inlet. A visual representation and a schematic layout of our setup can be found in Figure 4 and Figure 5, respectively. Once the fundamental arrangement was in place, the test rig was initiated, allowing water to circulate within a closed loop. To capture the vibration data from the CP, we employed four accelerometers. Two were affixed directly onto the pump casing, while the remaining two were positioned near the mechanical seal and the impeller. Each of these sensors channeled the vibration data of the pump through distinct recording paths. Subsequently, the signal underwent digitization at a signal monitoring station using a National Instruments 9234 device.

The accelerometer, model 622b01, operates within a frequency range of 0.4 to 10 kHz and boasts a sensitivity of 100 mV/g (10.2 mV/g(ms^−2^)) with an accuracy tolerance of ±5%. Complementing this, the data acquisition system, model NI9234, captures a broader frequency range up to 13.1 MHz and is designed with a generator that includes four analog input channels, each backed by a high-resolution 24 bit analog-to-digital converter (ADC), ensuring precise data representation. Furthermore, the CP vibration data were gathered at a steady rate of 1733 rpm, employing four accelerometers over approximately 300 s with a sampling frequency of 25.6 kHz. Channel 3, positioned axially to the CP, was specifically utilized for this data collection.

### 3.1. Dataset

In this study, we rigorously assess the proposed method’s generalizability by evaluating it across three distinct datasets. These datasets were meticulously gathered using the aforementioned data acquisition system, with each dataset compiled under varying pressure conditions that directly influence the method’s accuracy outcomes. The data are categorized into four classes: impeller fault (IF), mechanical seal hole (MSH), mechanical seal scratch (MSS), and normal (Norm) vibration signals, representing different CP health states. The composition of each dataset varies, with a unique count of samples contributing to the diversity of the data. Table 3 provides a detailed breakdown of the datasets, offering an illustrative snapshot of their structure and contents for further clarity.

### 3.2. Impeller Fault

Crevice corrosion is a prevalent factor leading to impeller failures, creating an irregular surface marred with a pattern of overlapping holes of varying sizes, resembling damage from insect consumption. Over time, the stress on the material around these holes can evolve into substantial cracks, escalating the risk of material fatigue and potentially resulting in a sudden and complete failure. In this study, an impeller exhibiting such corrosion-like defects was employed to collect vibration data indicative of this specific fault condition.

**Figure 6:**
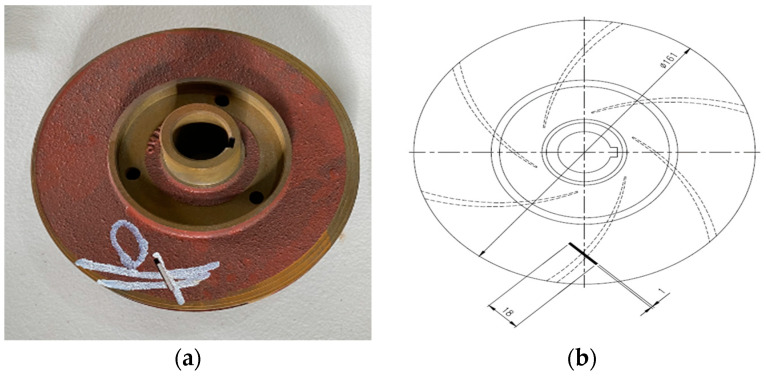
(**a**) Impeller fault and (**b**) its schematic diagram [45].

For the experiments, three cast iron impellers, each with a diameter of 161 mm, were used. Two of these impellers were brand new and in pristine condition. The third impeller, however, as illustrated in Figure 6, was intentionally altered to simulate a defect. This was performed by removing a section of metal, creating a flaw 2.5 mm wide, 18 mm long, and 2.8 mm deep.

### 3.3. Mechanical Seal Faults

Overpressure is the primary culprit in the failure of mechanical seals. During installation, mechanical seals rely on a spring or a set of springs to maintain the necessary contact between their rotating and stationary elements, preventing leaks in the pump. These springs are tuned to exert a certain compression force. If this force is breached, the seals are forced to bear an undue load of pressure. This excessive load can induce overheating, potentially vaporizing the vital lubricant film between the seals. Contamination, particularly by dirt, significantly endangers the seals. When the lubricating layer is compromised due to the overpressurization of the springs, entrapped dirt can inflict scratches, create holes, and render the seals brittle. Such early-stage failures can precipitate a catastrophic pump breakdown. This study deliberately introduced defects such as holes and scratches into the mechanical seals and monitored the associated vibration signals to devise strategies to preempt these premature failures.

#### 3.3.1. Mechanical Seal Hole

**Figure 7:**
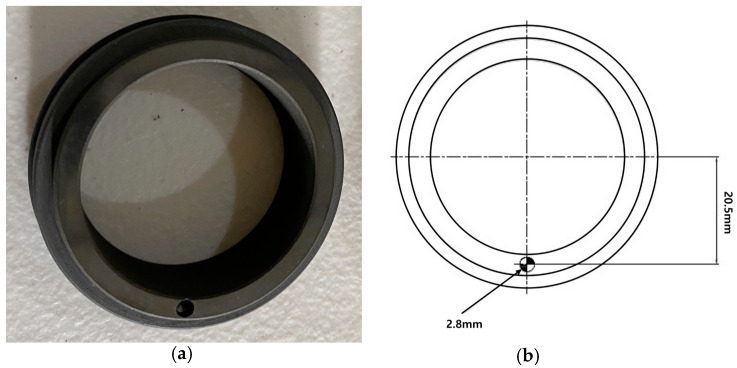
(**a**) Mechanical seal hole and (**b**) its schematic diagram [45].

The mechanical seal investigated in this study consists of two primary components: a rotating part and a stationary part, each with a diameter of 38 mm. As depicted in Figure 7, a defect was introduced in the form of a hole on the rotating part of the seal, while the stationary part was kept intact. The hole was precisely crafted with a diameter and depth of 2.8 mm each. This modified seal, exhibiting the hole defect, served as the basis for examining the subtle initial faults typically associated with mechanical seal hole imperfections.

#### 3.3.2. Mechanical Seal Scratch

**Figure 8:**
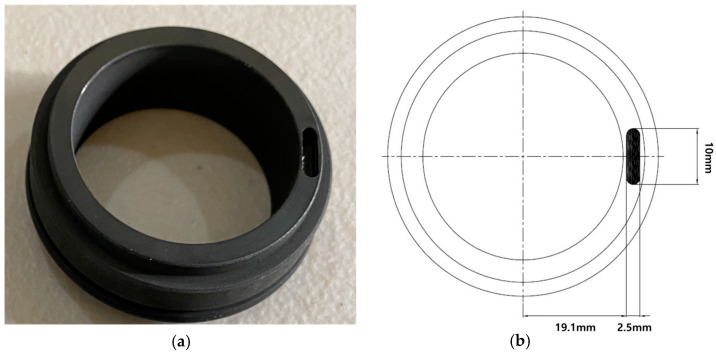
(**a**) Mechanical seal scratch and its (**b**) schematic diagram [45].

In the rotating section of the mechanical seal, a deliberate scratch was introduced, leaving the stationary section without any defects. The mechanical seal depicted in Figure 8 exhibits a significant defect in the form of a scratch, which is 2.5 mm wide, 10 mm long, and 2.8 mm deep.

**Figure 9:**
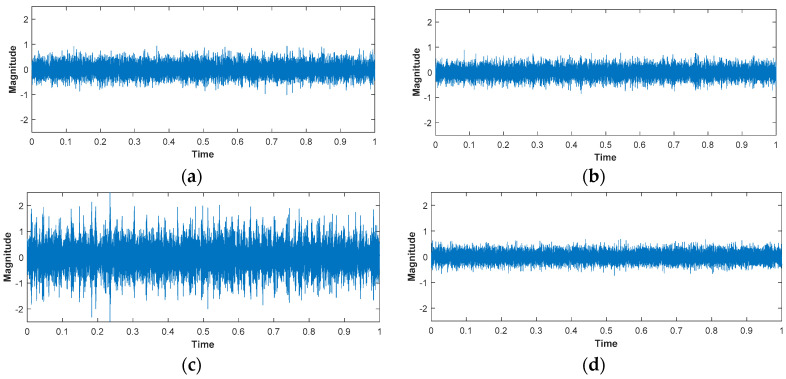
Time domain signal of (**a**) impeller fault, (**b**) mechanical seal hole, (**c**) mechanical seal scratch, and (**d**) normal.

Figure 9 illustrates the time domain vibration signal plots for impeller faults, mechanical seal holes, mechanical seal scratches, and normal conditions.

## 4. Results Discussion and Evaluation Metrics

The effectiveness of the proposed CP fault diagnosis method is thoroughly evaluated and presented in this section. This includes a detailed analysis of the method’s performance in accurately diagnosing faults, highlighting its strengths and potential areas for improvement. The evaluation is based on various metrics and tests, which are designed to rigorously assess the method’s reliability, accuracy, and overall diagnostic capabilities under different conditions and scenarios. This section aims to demonstrate the practical applicability and robustness of the CP fault diagnosis method in real-world settings.

### 4.1. Data Configuration for Training and Testing

To demonstrate the generalizability of the proposed method, it was tested across three distinct datasets, each collected under varying pressure conditions. Specifically, vibration signals in Dataset_1 were gathered at a pressure of 3.0 bars, comprising a total of 1247 samples. In Dataset_2, vibration signals were recorded at a pressure of 3.5 bar, encompassing 1324 samples, while Dataset_3 includes signals collected at a pressure of 4.0 bar, totaling 1281 samples. A crucial aspect of this process involved splitting these datasets in an 80/20 ratio for training and testing, respectively. This step is essential in training deep learning models, as it allows for the evaluation of the model on unseen data, prevents overfitting, and facilitates a more effective tuning of model parameters based on the model’s performance.

### 4.2. Evaluation Metrics

In this study, vibration signals were gathered using sensors, and a lowpass filter was employed to isolate fault-specific frequencies. Two parallel scalograms of these signals were generated, and a Sobel filter was applied to extract edges from the scalograms. These enhanced edge scalograms were then input into parallel CAEs for feature extraction. The features extracted from both CAEs were amalgamated into a hybrid feature pool. This pool was subsequently classified using an ANN. The results obtained from the proposed model demonstrated greater accuracy and generalizability compared to existing state-of-the-art models. To evaluate its performance, a set of measures, including accuracy, precision, recall, and the F1 score, were utilized. These measures, along with their mathematical formulations, are detailed below for a comprehensive performance comparison with reference methods [46].

(12) $$Acc=\left(\frac{TN+TP}{TN+TP+FN+FP}\right)100$$

(13) $$Pr=\left(\frac{TP}{TP+FP}\right)100$$

(14) $$Re=\left(\frac{TP}{TP+FN}\right)100$$

(15) $$F_{1}=2\times \frac{Pr\times Re}{Pr+Re}$$

where “Acc”, “Pr”, “Re”, and $“F_{1}”$ represent accuracy, precision, recall, and the F1 score, respectively. “TP” refers to a true positive, which is a positive sample correctly identified by the classifier. “TN” stands for a true negative, indicating the negative samples that the classifier has accurately identified. “FP” denotes a false positive, representing the positive samples that have been incorrectly identified by the classifier. Lastly, “FN” signifies a false negative, which is a negative sample that the classifier has incorrectly identified.

When the proposed method was applied to real-world industrial vibration data, it yielded impressive results: an accuracy of 99.24%, a precision of 99.37%, a recall of 99.23%, and an F1 score of 99.29%. These outcomes are detailed in Table 4, which compares the performance of the proposed method with those of reference methods. As illustrated in Table 5, the proposed method surpasses the reference methods in terms of classification accuracy. The superior performance of the proposed method can be attributed to its innovative approach, which leverages the strengths of two parallel scalograms and CAEs. This approach effectively combines extracted features into a unique hybrid feature pool, enhancing the overall performance across all evaluation metrics.

In 2022, Tran et al. introduced a method involving the collection of vibration signals and the creation of scalograms through a CWT. They developed a novel attention-based convolutional neural network for classifying these scalograms. This approach was specifically applied to induction motors, enabling the classification of vibration signals into five distinct classes: normal, outer ring fault, inner ring fault, misalignment, and broken rotor bar [47]. When the methodology outlined by Tran et al. [47] was implemented using our dataset, we observed the following results: an accuracy of 91.60%, a precision of 91.56%, a recall of 91.60%, and an F1 score of 91.43%.

In 2023, Manikandan et al. [48] conducted a pivotal study on vibration-based fault diagnosis in industrial mono-block centrifugal pumps. This research was centered around the development of an experimental setup, specifically designed to structure databases crucial for the advancement of machine learning algorithms. A key part of the study was the collection of vibration signals under standard operating conditions, ensuring the pump was in a defect-free, healthy state. The focus then shifted to identifying two primary defects: a broken impeller (B.I.) and a seal failure (S.F.). By introducing these faults sequentially into the pump, the researchers were able to capture the distinct vibration signals associated with each condition. These signals were then transformed into 2D images through an innovative image processing technique, facilitating a more detailed analysis. When this method was applied to our dataset, the results were noteworthy, achieving an accuracy of 93.20%, a precision of 93.32%, a recall of 93.23%, and an F1 score of 93.15%. This demonstrates the effectiveness of Manikandan et al.’s approach in accurately diagnosing faults in centrifugal pumps.

Utilizing three distinct datasets, Table 4 in our study presents a comprehensive comparison between the methodologies of Tran et al. and Manikandan et al. and contrasts these with our own approach. This comparison is extensive, encompassing all performance parameters for each fault class across the datasets. Furthermore, for an overview of the overall performance, Table 5 provides a comparative analysis of the average performance parameters. This includes a side-by-side evaluation of the two reference methods as well as our proposed method, offering a clear and detailed perspective on the effectiveness of each approach in fault classification.

**Figure 10:**
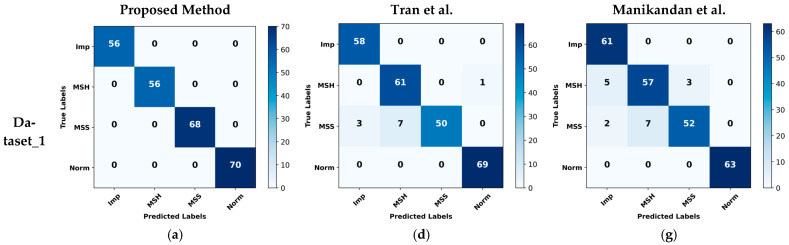

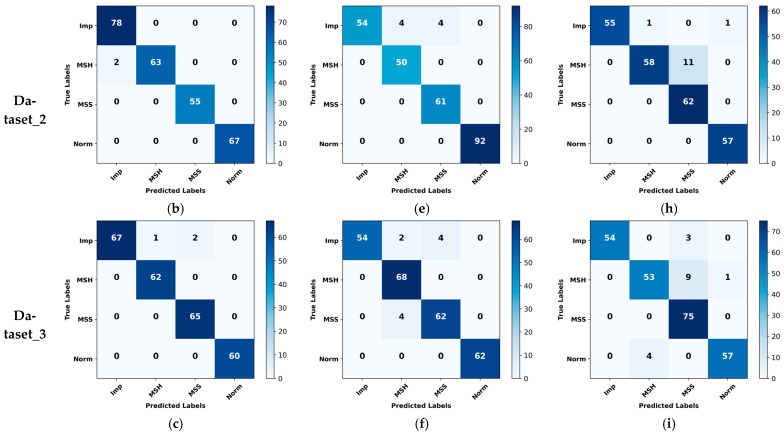
Confusion matrix comparison: Proposed method (**a**) Dataset_1, (**b**) Dataset_2, (**c**) Dataset_3; Tran’s method [47] (**d**) Dataset_1, (**e**) Dataset_2, (**f**) Dataset_3; Manikandan’s method [48] (**g**) Dataset_1, (**h**) Dataset_2, (**i**) Dataset_3.

In our study, Figure 10 presents a detailed analysis of confusion matrices derived from three distinct datasets, comparing the performance of our proposed method with two reference methodologies. These matrices serve as a clear visual representation of the classification accuracy across various fault conditions in the datasets. Notably, our method demonstrates a superior accuracy in comparison to the reference methods, as evidenced by the higher proportion of correct predictions (both true positives and true negatives) in the confusion matrices. For each dataset, the matrices provide insights into the specific areas where our method excels, particularly in reducing false positives and false negatives, which is critical for reliable fault diagnosis. This comparative analysis underscores the effectiveness of our approach, especially in terms of its robustness and precision in fault classification, thereby marking a significant advancement over existing techniques in the field of centrifugal pump fault diagnosis.

**Figure 11:**
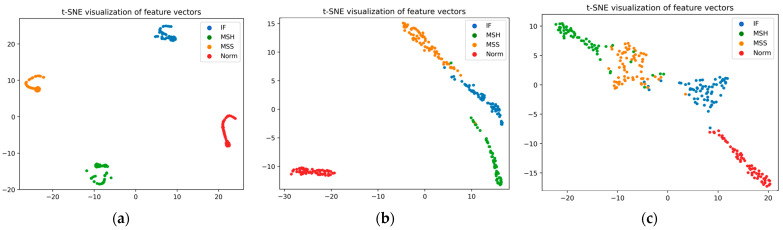
t-SNE feature plot representation of (**a**) proposed model, (**b**) Tran et al.’s model [47], and (**c**) Manikandan et al’s [48] model.

In our study, we employ t-SNE plots for a comparative analysis of feature spaces, providing a clear visual assessment of our proposed model’s performance relative to reference models. These plots effectively demonstrate the clustering patterns of data points, each symbolizing distinct fault conditions in centrifugal pumps. The t-SNE visualizations for each model distinctly showcase the distribution and separation of features within a complex, multidimensional space. While all models can easily separate faulty and healthy conditions, the reference models hardly separate different faulty conditions. In contrast, our proposed model not only classifies healthy and faulty states but also accurately classifies different faulty conditions. Figure 11 illustrates a graphical representation of these t-SNE plots, highlighting the superior classification capabilities of our proposed model compared to the reference models.

### 4.3. Discussion

In the comparative evaluation, our model, leveraging CNN and CWT scalograms for vibrational signal analysis, distinctly outperformed the methods employed by Tran et al. and Manikandan et al. In Dataset_1, our approach achieved optimal metrics with a score of 1.00 in accuracy, precision, recall, and the F1 score, demonstrating its superior diagnostic capability compared to the methods of Tran et al. and Manikandan et al., which achieved 0.955 and 0.932, respectively. This pattern of enhanced performance was consistent in Dataset_2, where our model scored 0.992, showcasing its precision and reliability, as opposed to the 0.969 and 0.946 achieved by the comparative studies. Furthermore, in Dataset_3, our model sustained its high performance with a score of 0.988, while Tran et al. and Manikandan et al.’s methods scored 0.960 and 0.933. This consistent achievement across different datasets underscores the robustness of our model and its proficiency in feature extraction and utilization for accurate fault classification, affirming its suitability for intricate industrial applications.

## 5. Conclusions

In conclusion, our study introduced a novel approach for fault diagnosis in centrifugal pumps, blending signal processing with deep learning. Starting with the collection and preprocessing of vibration signals, we applied a continuous wavelet transform and a Stockwell transform to generate detailed time–frequency scalograms, further refined by a Sobel filter for enhanced feature visibility. Central to our method are two convolutional autoencoders, each tailored to a specific scalogram type, facilitating the extraction of comprehensive and discriminative features. This innovative integration surpasses traditional methods, providing a holistic analysis of signal properties. The effectiveness of our approach is evidenced by its achievement of accuracies of 100%, 99.2%, and 98.8% across three datasets, using a two-layer artificial neural network for classification. The proposed method establishes a connection between traditional signal processing and advanced deep learning for diagnosing mechanical faults in CPs, holding substantial promise for predictive maintenance in industrial environments. However, the diagnostic capabilities of the proposed method have not been evaluated for hydraulic faults, such as cavitation, in the CP. In subsequent work, the method will be expanded to address both mechanical and hydraulic faults in the CP with reduced computational complexity.

## Figures and Tables

**Table 1:** Convolutional autoencoder layers and their output dimensions.

Encoder		Decoder	
Layer Name	Output Dimension	Layer Name	Output Dimension
Input	128 × 128 × 1	Dense	2048
Conv2D	64 × 64 × 16	Reshape	4 × 4 × 128
Conv2D	32 × 32 × 32	Conv2DTranspose	8 × 8 × 128
Conv2D	16 × 16 × 64	Conv2DTranspose	16 × 16 × 64
Conv2D	8 × 8 × 128	Conv2DTranspose	32 × 32 × 32
Conv2D	4 × 4 × 128	Conv2DTranspose	64 × 64 × 16
Flatten	2048	Conv2DTranspose	128 × 128 × 1

**Table 2:** Setting of hyperparameters in CAE and t-SNE.

Deep Learning Model	Hyperparameter	Value/Setting
Autoencoder	Batch size	32
	Hidden layers	11 (encoder 6, decoder 5)
	Optimizer type	Adam
	Loss function	MSE
	Learning rate	0.001
	Number of epochs	10
t-SNE	Number of components	2
	Perplexity	30
	Learning rate	200

**Table 3:** Number of samples with each health condition in each dataset.

CP’s Health Condition	Dataset_1 (3.0 BAR) No. of Samples	Dataset_2 (3.5 BAR) No. of Samples	Dataset_3 (4.0 BAR) No. of Samples
IF	304	307	303
MSH	311	312	313
MSS	315	291	346
Normal	317	414	319

**Table 4:** Comparative analysis of each class’s results: our proposed method versus those of Tran et al. [47] and Manikandan et al. [48].

Dataset_1																
	Accuracy				Precision				Recall				F1 Score			
**Model**	**IF**	**MSH**	**MSS**	**Normal**	IF	**MSH**	**MSS**	**Normal**	**IF**	**MSH**	**MSS**	**Normal**	**IF**	**MSH**	**MSS**	**Normal**
**Proposed**	**1.00**	**1.00**	**1.00**	**1.00**	**1.00**	**1.00**	**1.00**	**1.00**	**1.00**	**1.00**	**1.00**	1.00	**1.00**	**1.00**	**1.00**	**1.00**
Tran et al. [47].	1.00	0.98	0.83	1.00	0.95	0.90	1.00	0.99	1.00	0.98	0.83	1.00	0.97	0.94	0.91	0.99
Manikandan et al. [48].	1.00	0.87	0.85	1.00	0.90	0.89	0.95	1.00	1.00	0.88	0.85	1.00	0.95	0.88	0.90	1.00
**Dataset_2**																
**Proposed**	**1.00**	**0.96**	**1.00**	**1.00**	**0.97**	**1.00**	**1.00**	**1.00**	**1.00**	**0.96**	**1.00**	**1.00**	**0.98**	**0.98**	**1.00**	**1.00**
Tran et al. [47].	0.87	1.00	1.00	1.00	1.00	0.93	0.94	1.00	0.87	1.00	1.00	1.00	0.93	0.96	0.97	1.00
Manikandan et al. [48]	0.96	0.84	1.00	1.00	1.00	0.98	0.85	0.98	0.96	0.84	1.00	1.00	0.98	0.91	0.92	0.99
**Dataset_3**																
**Proposed**	**0.95**	**1.00**	**1.00**	**1.00**	**1.00**	**0.98**	**0.97**	**1.00**	**0.95**	**1.00**	**1.00**	**1.00**	**0.97**	**0.99**	**0.98**	**1.00**
Tran et al. [47].	0.90	1.00	0.93	1.00	1.00	0.92	0.94	1.00	0.90	1.00	0.94	1.00	0.95	0.96	0.94	1.00
Manikandan et al. [48]	0.94	0.84	1.00	0.93	1.00	0.93	0.86	0.98	0.95	0.84	1.00	0.93	0.97	0.88	0.93	0.96

**Table 5:** Average comparative analysis: our proposed method versus those of Tran et al. [47] and Manikandan et al. [48].

Dataset_1				
Model	Accuracy	Precision	Recall	F1 Score
Proposed	1.00	1.00	1.00	1.00
Tran et al. [47].	0.955	0.958	0.954	0.953
Manikandan et al. [48]	0.932	0.933	0.932	0.931
**Dataset_2**				
**Proposed**	**0.992**	**0.993**	**0.992**	**0.992**
Tran et al. [47].	0.969	0.966	0.967	0.965
Manikandan et al. [48]	0.946	0.953	0.951	0.949
**Dataset_3**				
**Proposed**	**0.988**	**0.988**	**0.989**	**0.988**
Tran et al. [47].	0.960	0.964	0.959	0.961
Manikandan et al. [48]	0.933	0.943	0.930	0.935

## Data Availability

The data are available upon the request.
